# Comparative Effectiveness of Ultrasound Guided Ozone (O_2_–O_3_) and Corticosteroid Injections for Sacroiliac Joint Dysfunction: A Multicenter Clinical Trial

**DOI:** 10.3390/jcm15062285

**Published:** 2026-03-17

**Authors:** Ridvan Isik, Muhammed Zahid Sahin, Emre Uzun, Ferhat Ege, Kemal Nas

**Affiliations:** 1 Department of Physical Medicine and Rehabilitation, Division of Pain Medicine, Sakarya Training and Research Hospital, 54290 Sakarya, Turkey; dr.ridvanisik@gmail.com; 2 Department of Physical Medicine and Rehabilitation, Faculty of Medicine, Sakarya University, 54290 Sakarya, Turkey; 3 Department of Physical Medicine and Rehabilitation, Sakarya Training and Research Hospital, 54290 Sakarya, Turkey; ftremreuzun@gmail.com; 4 Department of Physical Medicine and Rehabilitation, Division of Pain Medicine, Diyarbakır Gazi Yaşargil Training and Research Hospital, 21010 Diyarbakır, Turkey; dr.ege_ferhat@hotmail.com; 5 Department of Physical Medicine and Rehabilitation, Division of Pain Medicine, Faculty of Medicine, Sakarya University, 54290 Sakarya, Turkey

**Keywords:** sacroiliac joint, low back pain, ozone, corticosteroids, ultrasonography

## Abstract

**Background/Objectives**: Sacroiliac joint (SIJ) dysfunction is a common yet frequently underdiagnosed cause of chronic low back pain. This study aimed to compare the clinical effectiveness of ultrasound-guided corticosteroid and ozone injections in patients with chronic low back pain due to SIJ dysfunction. **Methods**: This comparative clinical study included 64 patients with chronic sacroiliac joint (SIJ) dysfunction who received ultrasound-guided SIJ injections with either corticosteroid (n = 31) or ozone (n = 33). Participants had a mean age of 45.0 ± 7.7 years, and the sex distribution was 42/22 (female/male). Pain intensity was assessed using the Numeric Rating Scale (NRS), disability using the Oswestry Disability Index (ODI), and quality of life using the Short Form-12 Physical (PCS) and Mental (MCS) Component Summary scores. Outcomes were evaluated at baseline, 3 months, and 6 months. Longitudinal changes were analyzed using two-way repeated-measures ANOVA (group × time) with Bonferroni-adjusted post hoc comparisons. Effect sizes were calculated using Cohen’s d. Normality and homoscedasticity were assessed (Shapiro–Wilk and Levene tests), and baseline comparisons were performed using appropriate parametric or non-parametric tests. **Results**: Both treatments significantly improved pain, disability, and quality of life at 3 months (*p* < 0.01). However, improvements were significantly greater and more durable in the ozone group across all outcomes at both 3 and 6 months (*p* < 0.01). At 6 months, between-group differences favored ozone for NRS (mean difference −2.81; Cohen’s d = −2.36), ODI (−6.05; d = −1.46), SF-12 PCS (+4.24; d = 1.24), and SF-12 MCS (+4.22; d = 0.83). A ≥50% pain reduction was achieved at 3 months in 97.0% of ozone-treated patients versus 45.2% of corticosteroid-treated patients (*p* < 0.01) and persisted at 6 months in 18.2% and 0% of patients, respectively (*p* < 0.05). The magnitude of improvement in the ozone group exceeded commonly reported Minimal Clinically Important Difference (MCID) thresholds for chronic low back pain outcomes, supporting clinical relevance. **Conclusions**: Ultrasound-guided ozone injection provided greater and more durable improvements in pain relief, functional status, and quality of life compared with corticosteroid injection in patients with SIJ dysfunction.

## 1. Introduction

Low back pain (LBP) is one of the most prevalent worldwide health issues and imposes a considerable cost on society. It is estimated that approximately 70–84% of individuals experience low back pain at some point during their lifetime, with reported annual prevalence rates ranging from 15% to 65% and point prevalence between 30% and 33% [1]. In Türkiye, low back pain is similarly widespread, with lifetime prevalence reported between 44% and 79%, point prevalence around 19.7–20.1%, and an annual prevalence of approximately 36% [1]. Beyond its high prevalence, low back pain is a leading cause of disability, reduced work productivity, and increased healthcare utilization [1].

LBP can develop from a variety of anatomical components, including muscles, intervertebral discs, fascia, and facet joints. Sacroiliac joint (SIJ) pain is another frequent source of LBP, accounting for about 10–38% of all cases [2].

The etiology of sacroiliac joint pain involves multiple factors, including age-related degenerative changes, joint laxity, and trauma [3]. Since there are no reliable clinical and radiological tests, a combination of provocative tests and diagnostic injections should be employed to reach this diagnosis [4]. The current clinical treatment of SIJ dysfunction is composed of NSAIDs, physical therapy, manual therapy, therapeutic injections, and surgery.

Corticosteroid injections are commonly employed in the treatment of SIJ complex pain [5]. Intra-articular injections, utilized for diagnostic and therapeutic purposes, exhibit limited accuracy due to anatomical variability and the extravasation of injectate beyond the joint space. Current evidence indicates that both intra- and extra-articular corticosteroid injections can offer short-term pain relief in suitably selected patients [6]. The findings highlight the heterogeneous characteristics of SIJ complex pain and underscore the necessity of addressing both articular and periarticular targets in treatment approaches.

Ozone is a gas that has anti-inflammatory, anti-microbial, and immunomodulatory functions. It displays its action via enhancing tissue oxygenation and circulation, removing inflammatory mediators from the joint space, normalizing intracellular antioxidants and nitric oxide levels, and decreasing superoxide production [7]. Recently, Ozone therapy has been suggested as a potential alternative to conventional treatments for the relief of pain and functional impairment caused by musculoskeletal conditions, and this treatment seems to be effective in the literature [7,8].

To date, it has been proven by investigations that SIJ injections using various types of injectate, including steroids, prolotherapy, platelet-rich plasma, and neuroablative techniques, are efficacious in managing SIJ pain [9,10,11,12]. Yet, to the best of our knowledge, there is no research that compares ozone injection to corticosteroid injection in patients experiencing pain in the SIJ complex. In light of this research gap, this study aimed to compare pain, functional status, and quality of life among patients with SIJ dysfunction who received ozone or corticosteroid injections.

## 2. Materials and Methods

### 2.1. Research Design and Population

This retrospective, multi-center, cohort study was carried out at two training and research hospital outpatient pain clinics from October 2022 to June 2024. The present study was approved by a local ethical committee (approval date: 27 June 2024) and performed in conformity with the Declaration of Helsinki and its subsequent amendments (Ethics number: E-71522473-050.04-372990-200). After obtaining ethics committee approval, the files of patients who underwent intra-articular steroid or ozone injection for SIJ dysfunction were reviewed from the hospital registration system. Treatment selection was based on routine clinical practice and patient preference after detailed information was provided regarding both options. Corticosteroid injections were fully covered by the public health system, whereas ozone therapy required payment by the patient.

Inclusion Criteria: Patients who had experienced low back pain for at least three months and were later diagnosed with SIJ dysfunction in our pain clinic after conducting a thorough clinical and physical examination, as well as radiologic assessments. Other inclusion criteria were having intractable pain at the SIJ with a numeric rating scale (NRS) of four or higher despite conservative treatment for SIJ dysfunction and being between the ages of 18 and 65 [13].

All patients who applied to our pain clinic with low back pain underwent a clinical evaluation for diagnosis. To diagnose SIJ dysfunction, provocative tests such as FABER, Gaenslen, tight thrust, compression, and distraction were implemented for patients with pain around the SIJ, and afterward, those who tested positive for at least three of them received an ultrasound-guided SIJ injection using local anesthesia (1 mL intra-articular, 1 mL peri-articular 1% lidocaine) as the diagnostic standard [14]. Participants with at least a 75% reduction in pain intensity on the NRS after the injection were accepted into the study. Images were taken to rule out further potential causes, such as sacroiliitis and lumbar spinal conditions.

Exclusion Criteria: Patients who had received a therapeutic SIJ injection within the previous three months, having sacroilitis, patients having a history of lumbar surgery, systemic and/or local infections, malignancy, bleeding diathesis, acute fracture, a known history of any psychiatric issues, and glucose-6-phosphate dehydrogenase deficiency (G6PDD), patients without demographic and clinical data, or direct radiographs.

Sample size calculation was performed using G*Power software version 3.1.9.7 (Heinrich-Heine-Universität, Düsseldorf, Germany) according to the results of a previous study [15]. In that study, the mean [± standard deviation (SD)] VAS score at 3 months was 3.43 ± 1.38. Considering the VAS score as the primary outcome measure at month 3, a sample size of 30 patients in each group was determined to detect a 30% between-group difference, with a significance level of 0.05, corresponding to a 95% confidence interval, and a power of 80%.

All patients who participated in the research gave their verbal and written informed consent.

In this study, demographic data, symptom duration, the NRS for pain scoring, the Short Form-12 Health Survey (SF-12) for quality of life, the Oswestry Disability Index (ODI) for functional status, and the presence of any complications in the post-injection period were collected by examining the hospital registration system.

### 2.2. Groups

Patients were categorized into two groups: Group CS (Corticosteroid) and Group O (Ozone). Patients in Group CS received a total of 2 mL of solution mixture, with 1 mL intra-articular and 1 ml peri-articular injection. The solution mixture was 1 mL (4 mg) dexamethasone + 1 mL 0.5% bupivacaine. Patients in Group O received 25 μg/mL-2 mL ozone administered as 1 mL intra-articular and 1 mL periarticular injections.

Due to the short half-life of ozone (about 45 min at 20 °C), we produced it fresh using an ozone generator (TURKOZONE Blue-S—Istanbul, Turkey) connected to a pure oxygen source and used it immediately for the patient. Ozone generators use oxygen through high-voltage tubes in the output range of 4000–14,000 and produce an O_2_–O_3_ mixture with concentration ranges up to 5%.

Patients in the corticosteroid group received a single injection, whereas patients in the ozone group received a total of three injection sessions, once a week. We provided the patients with a standardized home exercise program consisting of SIJ-targeted stretching, self-mobilization techniques, and spinal-core stabilization exercises. Stretching exercises focused on the hip flexors, hamstrings, piriformis, and lumbar paraspinal muscles. Self-mobilization techniques included gentle pelvic tilting and controlled lumbopelvic movements within a pain-free range. Core stabilization exercises emphasized activation of the transversus abdominis and multifidus muscles, including abdominal drawing-in maneuvers and bridging exercises. Patients were instructed to perform the exercises as 3 sets of 10 repetitions daily. The exercises were demonstrated during the outpatient visit, and patients received verbal and written instructions for home application.

The primary outcome measure of the study was the change in the NRS for pain following intervention. Secondary outcome measures were changes in ODI and SF-12 scores and the presence of complications.

### 2.3. Interventional Procedure

In both centers, the procedures were performed in a special injection room under ultrasound guidance (HM70 EVO, Samsung Healthcare, Seoul, South Korea) [16], a commercially available high-resolution musculoskeletal ultrasound system widely used in clinical practice, by qualified pain medicine physicians (RI, FE). Patients underwent standard monitoring throughout the procedure, including electrocardiography, non-invasive blood pressure, and peripheral oxygen saturation, and an IV catheter was placed pre-procedure as a precaution.

**Figure 1:**
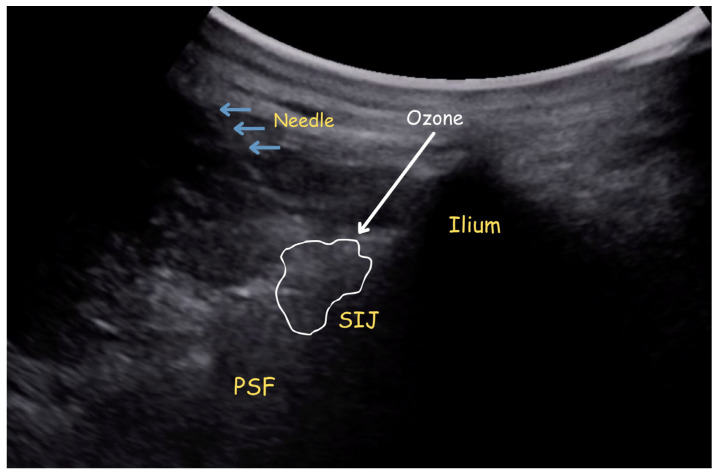
Ultrasound image demonstrating the needle trajectory, ozone distribution, and relevant anatomical structures during sacroiliac joint injection. PSF: posterior sacral foramen, SIJ: sacroiliac joint.

The patients were required to lie down in a prone position, and subsequently, a curvilinear US transducer (3–16 MHz) connected to an ultrasound system (HM70 EVO, Samsung Healthcare, Seoul, South Korea) was used to detect SIJ. The US probe was disinfected and enclosed in a sterile bag, and then the patient’s skin was sterilized with iodopovidone. As the sonographic interface, a customized sterile gel was utilized. First, the probe was placed on the spinal midline in the transverse axis, the lumbar spinous processes were determined, and then it was shifted slightly caudal and lateral to detect the sacroiliac joint complex. Once the target site was identified, a 22-gauge spinal needle was advanced from medial to lateral at the S1 foramen level into the peri-articular structures, and at the S2 foramen level, the injection was administered into the joint (Figure 1). All participating centers followed the same institutional protocols for ultrasound-guided SIJ injections and outcome assessments.

Patients were followed up in the observation room for thirty minutes after the injection for the presence of any possible complications. Patients with no complications were discharged with recommendations and asked to return for follow-up visits after 3 and 6 months.

During the follow-up period, patients were permitted to use commonly prescribed oral analgesics as needed, including nonsteroidal anti-inflammatory drugs (NSAIDs) such as ibuprofen (up to 1200–1800 mg/day), diclofenac (up to 100–150 mg/day), or naproxen (up to 500–1000 mg/day), as well as paracetamol (up to 3–4 g/day), in accordance with standard therapeutic dosing recommendations. Analgesic use was not protocol-restricted but followed routine outpatient prescribing practices. No additional interventional procedures were performed during the follow-up period.

Adverse events such as transient pain exacerbation or symptom recurrence were managed conservatively, and no serious intervention-related complications were identified in the medical records. Given the retrospective design, no protocol-driven monitoring was implemented between baseline and the 3-month follow-up. However, any adverse events or unscheduled visits were documented in the hospital information system and reviewed retrospectively.

### 2.4. Assessment Parameters

The NRS, the ODI, and the SF-12 were utilized before the procedure and in the 3rd and 6th months following the procedure.

The NRS is a commonly employed measure for evaluating and monitoring pain intensity. It is an 11-point rating, with 0 representing no pain and 10 representing the worst pain imaginable, and the patient is asked to rate his or her pain on the scale from 0 to 10. The NRS is a valid and reliable instrument for assessing pain intensity, demonstrating excellent test–retest reliability and strong validity across clinical studies. In patients with musculoskeletal pain, including osteoarthritis, the NRS has demonstrated excellent test–retest reliability with an intraclass correlation coefficient (ICC) of 0.95 (95% CI 0.93–0.96). The standard error of measurement (SEM) was reported as 0.48, and the minimal detectable change (MDC) as 1.33 points [17].

The ODI was used to assess the participants’ degree of disability owing to low back pain. The measure examines 10 parameters, including pain intensity, personal care, lifting, walking, sitting, standing, sleeping, sex life, social life, and travel. Each item obtains a score between 0 (better) and 5 (worse). Each participant’s ODI score was recorded and then converted to a 0–100 scale.

SF-12 has been used to evaluate the quality of life. This measure is a health-related quality-of-life questionnaire consisting of twelve items that examine physical and mental health by measuring eight health categories. The ODI and SF-12 have been culturally adapted and validated for use in the Turkish population, demonstrating good reliability, internal consistency, and construct validity in patients with musculoskeletal conditions, including low back pain [18,19]. The Turkish version of the ODI has demonstrated excellent test–retest reliability (ICC = 0.938, *p* < 0.001) and high internal consistency (Cronbach’s alpha = 0.918 and 0.895 at two assessments). Construct validity was supported by strong correlations with the Roland-Morris Disability Questionnaire (r = 0.815, *p* < 0.001) [18]. The Turkish version of the SF-12 has demonstrated acceptable internal consistency (Cronbach’s alpha = 0.73 for PCS-12 and 0.72 for MCS-12) and good test–retest reliability over a two-week interval (ICC = 0.73 for PCS-12 and 0.72 for MCS-12). Strong correlations were observed with the corresponding SF-36 components (PCS r = 0.93; MCS r = 0.96; *p* < 0.001), supporting criterion validity [19].

### 2.5. Statistical Analyses

Statistical analyses were performed using IBM SPSS Statistics for Windows, version 31.0 (IBM Corp., Armonk, NY, USA). The assumptions of normality and sphericity were assessed prior to repeated-measures ANOVA. Normality of residuals was evaluated using the Shapiro–Wilk test and visual inspection of residual plots, while sphericity was tested using Mauchly’s test. When sphericity was violated, Greenhouse–Geisser correction was applied. Prior to performing parametric analyses, the assumptions of normality and homogeneity of variances were evaluated. The normality of continuous variables was assessed using the Shapiro–Wilk test, and homogeneity of variances was examined using Levene’s test. Normally distributed data were expressed as mean ± standard deviation, whereas non-normally distributed data were presented as median (interquartile range). Categorical variables were reported as numbers and percentages. Subgroup analyses according to sex were not performed, as the study was not powered to detect sex-specific differences.

Baseline (cross-sectional) comparisons between the corticosteroid and ozone groups were conducted using the independent samples *t*-test or the Mann–Whitney U test, as appropriate. Categorical variables were compared using the chi-square test, and Fisher’s exact test was applied when necessary.

Changes in NRS, ODI, and SF-12 (PCS and MCS) over time were analyzed using two-way repeated-measures ANOVA (group × time). When significant effects were detected, Bonferroni-adjusted post hoc (simple effects) comparisons were performed to compare groups at each time point.

## 3. Results

**Figure 2:**
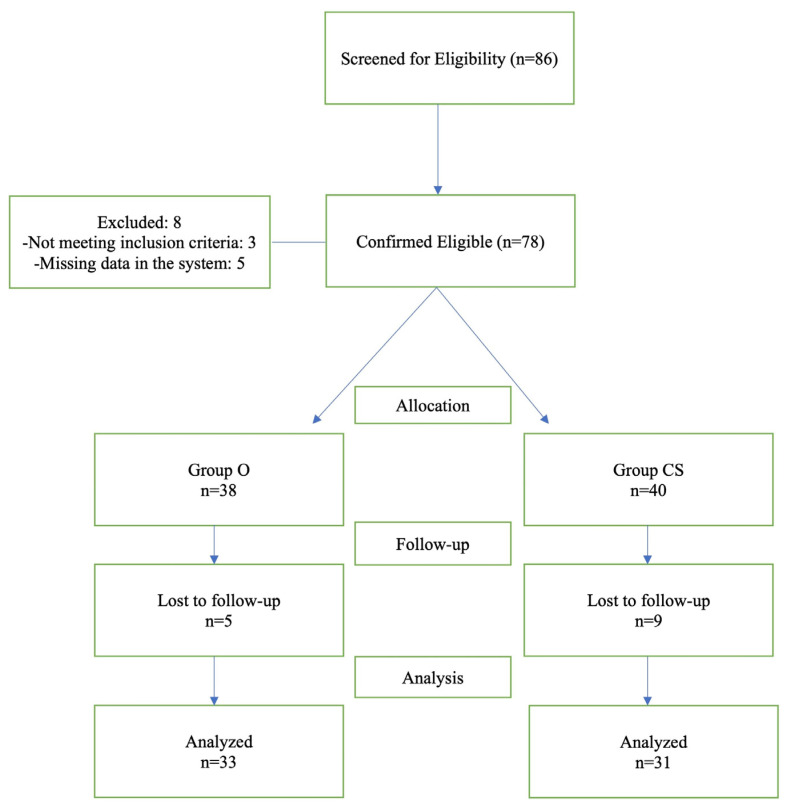
Patient selection flow chart.

A total of 86 patients were screened, of whom 64 were included in the analysis. Certain cases were omitted from the analysis due to missing data in hospital records. The study population consisted of 31 patients in the corticosteroid group and 33 patients in the ozone group (Figure 2).

Baseline demographic characteristics were comparable between groups. There were no significant differences in age (44.84 ± 6.64 vs. 45.15 ± 8.60 years, *p* = 0.872), sex distribution (female/male: 21/10 vs. 21/12, *p* = 0.730), or body mass index (28.55 ± 2.41 vs. 28.12 ± 2.16 kg/m^2^, *p* = 0.460). Symptom duration was also similar between the corticosteroid and ozone groups (median 12.0 [IQR 8] vs. 10.0 [IQR 7] months, *p* = 0.471). Marital status did not differ between groups (*p* = 0.750). These findings indicate that the groups were well matched before treatment, allowing reliable retrospective comparisons (Table 1).

**Figure 3:**
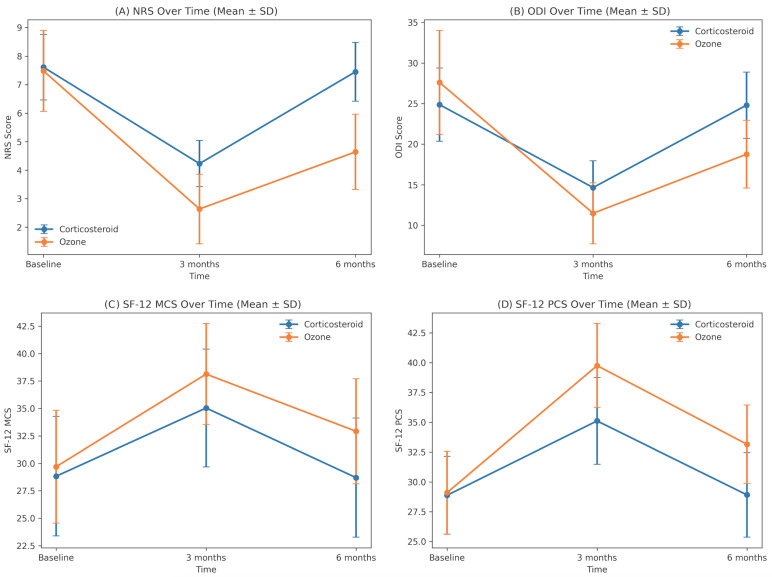
Changes in mean NRS, ODI, SF-12 MCS and SF-12 PCS over time in the ozone and corticosteroid groups.

Pain intensity assessed by NRS differed significantly between groups over time. Baseline NRS scores were similar between the ozone and corticosteroid groups (7.48 ± 1.42 vs. 7.61 ± 1.15, *p* = 0.693). At 3 months, the ozone group showed a markedly greater reduction in pain compared with the corticosteroid group (2.64 ± 1.22 vs. 4.23 ± 0.81, *p* < 0.001). This between-group difference persisted at 6 months, with lower NRS scores in the ozone group (4.64 ± 1.32 vs. 7.45 ± 1.03, *p* < 0.001) (Table 2, Figure 3).

Disability assessed by ODI showed significant between-group differences over time. Baseline ODI scores were similar in the ozone and corticosteroid groups (27.61 ± 6.41 vs. 24.87 ± 4.51, *p* = 0.054). At 3 months, the ozone group had significantly lower ODI scores than the corticosteroid group (11.48 ± 3.76 vs. 14.65 ± 3.29, *p* < 0.001). This difference persisted at 6 months, with ODI remaining lower in the ozone group (18.76 ± 4.17 vs. 24.81 ± 4.09, *p* < 0.001) (Table 2, Figure 3).

At 6 months, between-group differences in NRS (−2.81; Cohen’s d = −2.36), ODI (−6.05; d = −1.46), and SF-12 PCS/MCS (+4.24/+4.22; d = 1.24/0.83) met or exceeded established Minimal Clinically Important Difference (MCID) thresholds, supporting both the statistical and clinical relevance of the observed effects.

Physical component quality-of-life scores (SF-12 PCS) showed significant between-group differences over time. Baseline PCS were similar in the ozone and corticosteroid groups (29.10 ± 3.47 vs. 28.88 ± 3.27, *p* = 0.803). At 3 months, the ozone group had significantly higher PCS than the corticosteroid group (39.75 ± 3.52 vs. 35.11 ± 3.64, *p* < 0.001). This difference persisted at 6 months, with PCS remaining higher in the ozone group (33.15 ± 3.29 vs. 28.91 ± 3.54, *p* < 0.001) (Table 2).

Mental component quality-of-life scores (SF-12 MCS) showed significant between-group differences over time. Baseline MCS were similar in the ozone and corticosteroid groups (29.70 ± 5.14 vs. 28.83 ± 5.45, *p* = 0.515). At 3 months, the ozone group had a significantly higher MCS than the corticosteroid group (38.13 ± 4.60 vs. 35.04 ± 5.36, *p* = 0.016). This difference persisted at 6 months, with MCS remaining higher in the ozone group (32.92 ± 4.79 vs. 28.70 ± 5.42, *p* = 0.002) (Table 2).

**Figure 4:**
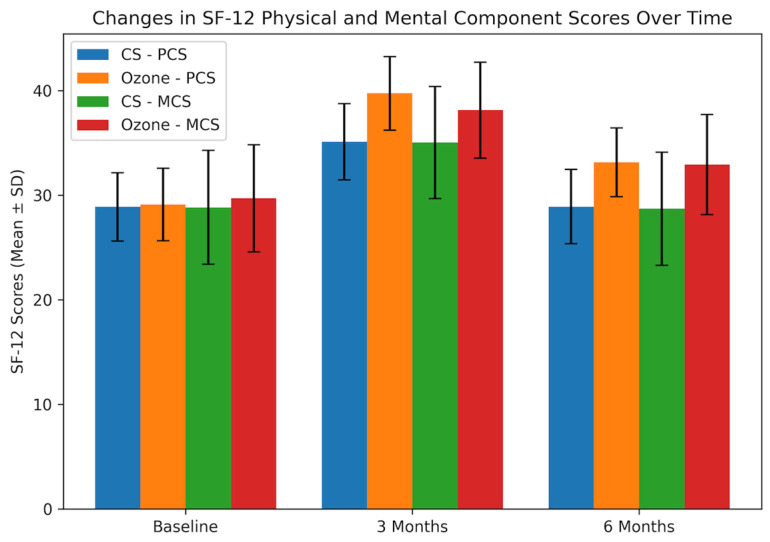
Changes in quality-of-life outcomes over time in the corticosteroid (CS) and ozone groups. Physical Component Score (PCS) and Mental Component Score (MCS) of the SF-12 are presented as mean ± standard deviation at baseline, 3 months, and 6 months.

When within-group changes were examined, both treatment groups showed significant short-term improvements in pain, disability, and quality-of-life scores (all *p* < 0.001, Table 3). From baseline to 3 months, NRS and ODI scores decreased, and SF-12 PCS and MCS increased significantly in both the corticosteroid and ozone groups, with larger mean changes in the ozone group. However, from baseline to 6 months, these improvements were maintained only in the ozone group (all *p* < 0.001); in the corticosteroid group, all scores at 6 months returned to values close to baseline (Table 3). The changes in quality-of-life outcomes (SF-12 PCS and MCS) over time are illustrated in Figure 4.

**Figure 5:**
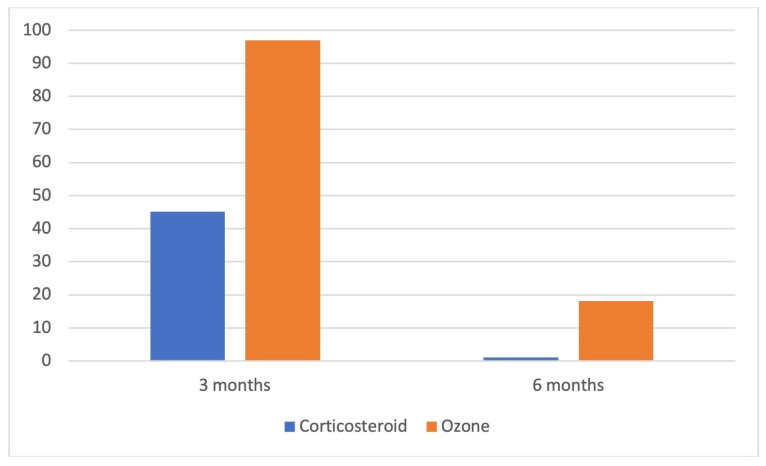
Proportion of patients achieving ≥50% reduction in NRS pain scores at 3 and 6 months.

A ≥50% reduction in NRS pain score from baseline at 3 months was achieved in 32 (97.0%) patients in the ozone group and 14 (45.2%) in the corticosteroid group (*p* < 0.001). At 6 months, this level of pain reduction persisted in 6 patients (18.2%) in the ozone group, whereas no patients in the corticosteroid group achieved this outcome (*p* = 0.013) (Figure 5).

No serious adverse events were observed in either group during the 6-month follow-up period. Transient procedural site pain was reported in 2 (6.5%) patients in the corticosteroid group and 3 (9.1%) patients in the ozone group.

## 4. Discussion

This clinical study compared the clinical effectiveness of ultrasound-guided corticosteroid and ozone injections in patients with chronic low back pain caused by SIJ dysfunction. The findings demonstrate that although both treatments provide meaningful short-term improvements, ozone therapy offers substantially greater and more durable benefits across all evaluated domains, including pain intensity, functional disability, and both physical and mental quality of life. In addition to statistical significance, the observed improvements with ozone therapy exceeded established MCID thresholds for pain, disability, and quality of life, suggesting that these changes are likely to be meaningful from the patient’s perspective. These results provide important new evidence to the evolving literature on minimally invasive interventions for SIJ dysfunction.

Previous studies have reported that corticosteroid injections provide meaningful short-term relief in SIJ pain but offer limited durability, with clinical benefits often diminishing within several weeks to a few months. In a recent randomized clinical trial by Sayed Fargaly et al. [20], which compared ultrasound-guided intra-articular corticosteroid injection with platelet-rich fibrin (PRF) in 94 patients with SIJ dysfunction. Although the steroid group experienced greater immediate post-procedure pain reduction, this benefit was short-lived. PRF produced significantly lower VAS scores at 1 week, 1 month, 3 months, and 6 months. Similarly, Das et al. [21] reported that although both corticosteroids and cryoneurolysis significantly reduced SIJ pain, cryoneurolysis yielded markedly greater and longer-lasting analgesia, with far higher proportions of patients achieving ≥50% pain reduction at 1, 3, and 6 months. These findings emphasize that while steroids can be effective initially, their therapeutic benefit is relatively short, and alternative modalities may offer more durable outcomes. It was also demonstrated in a prospective case series by Liliang et al. [22] that triamcinolone sacroiliac injections produced prolonged pain relief in a substantial proportion of patients. Approximately two-thirds experienced improvement lasting about 37 weeks. However, the remaining third reported only brief relief of around 4 weeks, particularly among those with a history of lumbar fusion. This indicates that the duration of steroid-induced pain relief can be strongly influenced by underlying biomechanical alterations and individual patient characteristics. Our findings are consistent with these observations. Although the corticosteroid group demonstrated significant improvement at 3 months, this effect was not sustained at 6 months, when pain and functional scores returned close to baseline.

In contrast, while ozone therapy has been studied in other musculoskeletal and spine-related conditions, no prior research, to the best of our knowledge, has specifically evaluated its use in the SIJ, and direct comparisons with corticosteroids in this region have not been previously reported. A key contribution of our study is the demonstration of a sustained therapeutic effect from ozone injection at 6 months. Patients receiving ozone maintained improvements in pain, disability, and quality of life over time, and 18.2% continued to achieve ≥50% pain reduction at 6 months, whereas no patients in the corticosteroid group achieved this outcome.

Ozone is a potent oxidizing agent that induces a controlled, transient oxidative stress through the generation of reactive oxygen species and lipid oxidation products. This stimulus activates endogenous antioxidant defenses, increases anti-inflammatory cytokines and growth factors, and suppresses pro-inflammatory cytokines, COX-2, and proteolytic enzymes, thereby contributing to its analgesic and anti-inflammatory effects [23].

The sustained benefits of ozone therapy are consistent with results in lumbar disc herniation and knee osteoarthritis, suggesting that similar biological mechanisms may underlie its effectiveness in SIJ dysfunction. This is supported by a recent meta-analysis by Chang et al. [24], which evaluated the effectiveness of intradiscal ozone injections in patients with lumbar disc herniation. Across multiple studies, intradiscal ozone demonstrated superior pain reduction and higher treatment success rates compared with steroid injections and conventional medications at both short-term (<6 months) and long-term (≥6 months) follow-up. Notably, long-term outcomes were comparable to those achieved with microdiscectomy, indicating a level of efficacy approaching that of surgical intervention. Consistent with these results, a randomized double-blind controlled trial by Ercalik et al. [25] found that intradiscal ozone therapy, whether administered alone or combined with periforaminal steroid injection, produced significant improvements in pain, disability, and quality of life at 1 and 6 months. Importantly, no significant differences were observed between the groups at any time point. The absence of any added benefit from steroid co-administration suggests that ozone alone can be sufficient to achieve meaningful clinical improvement. A double-blind randomized clinical trial by Babaei-Ghazani et al. [26] in knee osteoarthritis patients also supports the longer-lasting effects of ozone compared with corticosteroids. They showed that both treatments resulted in significant short-term improvements. However, at 3 months, reductions in VAS and WOMAC scores were significantly greater in the ozone group. Although the range of motion and joint effusion did not differ between groups, only the ozone group demonstrated a significant reduction in effusion on ultrasound. These findings highlight that ozone not only provides longer-lasting symptomatic relief than corticosteroids but may also exert additional local effects on joint inflammation.

In our current study, a clear internal consistency was observed across the pain, disability, and quality-of-life measures. In both groups, early improvements in NRS scores at 3 months were accompanied by reductions in ODI and increases in SF-12 PCS and MCS. However, at the 6-month mark, the corticosteroid group’s return to baseline pain levels was accompanied by a regression of functional and quality-of-life scores. This parallel decline supports the interpretation that the therapeutic effect of corticosteroids is transient and insufficient for sustained improvement. Conversely, the ozone group showed persistent improvements across all outcome domains at 6 months. The alignment between long-term pain reduction, physical function, and mental well-being in the ozone group strengthens the validity of the treatment effect and underscores the multidimensional benefit of ozone therapy.

Ozone therapy, owing to its longer-lasting efficacy, may reduce treatment burden while minimizing the risks associated with repeated corticosteroid use. Moreover, its favorable safety profile and non-pharmacologic mechanism make ozone especially suitable for individuals with comorbidities or contraindications that limit corticosteroid administration. Repeated administration of corticosteroid injections may be associated with local effects such as tissue atrophy and cartilage degeneration, as well as systemic effects including transient hyperglycemia and hypothalamic–pituitary–adrenal axis suppression. These considerations are particularly relevant in patients with comorbidities and should be considered when planning long-term management [27].

The effective and longer-lasting improvements observed with ozone therapy underscore the need for further investigation through well-designed prospective randomized controlled trials to validate these findings and address the inherent limitations of retrospective analyses. Future research should also focus on elucidating the biological mechanisms responsible for ozone’s prolonged therapeutic effects compared with corticosteroids, as well as determining optimal dosing parameters through dose–response studies. Additionally, incorporating imaging modalities such as MRI and exploring biochemical or inflammatory markers may help identify patient subgroups most likely to benefit from ozone treatment.

Ozone therapy may also be considered as part of a multimodal treatment strategy for sacroiliac joint dysfunction. Combining ozone injection with structured physical therapy, core stabilization exercises, or rehabilitation programs may potentially enhance functional recovery and prolong therapeutic benefits. However, the additive or synergistic effects of such combined approaches remain to be established. Future prospective studies are warranted to evaluate the safety, effectiveness, and optimal sequencing of ozone therapy within comprehensive pain management protocols.

To further contextualize the clinical relevance of these findings, the magnitude of the observed changes was interpreted in relation to commonly reported MCID thresholds. For chronic low back pain, a reduction of approximately 2 points on the NRS and an improvement of around 10 points on the ODI are generally considered clinically meaningful [28]. Similarly, changes of approximately 3–5 points in the SF-12 PCS and SF-12 MCS have been suggested to represent clinically important improvements in health-related quality of life [29]. In the present study, the mean reductions in NRS and ODI scores and the improvements observed in SF-12 PCS and MCS, particularly in the ozone group at both 3 and 6 months, met or exceeded these commonly accepted MCID thresholds. This indicates that the observed benefits are not only statistically significant but also clinically meaningful from the patient’s perspective.

This study has several important strengths. SIJ-related pain was rigorously confirmed using a standardized diagnostic approach, with patients required to test positive on at least three provocation tests and subsequently undergo an ultrasound-guided diagnostic SIJ injection using combined intra-articular and peri-articular local anesthesia, thereby increasing diagnostic accuracy [14]. Procedural accuracy and consistency were further enhanced by the use of ultrasound guidance. Additionally, the multicenter design improves the generalizability of the findings. This is the first study to directly compare ozone and corticosteroid injections for SIJ-related low back pain, providing novel evidence supporting the clinical effectiveness of ozone therapy in this patient population.

Several limitations of this study should also be acknowledged. First, the retrospective design introduces potential selection bias, missing data, and limited control over confounding factors. Second, although the sample size was sufficient to detect differences between groups, it may still restrict the broader applicability of the results. Third, the absence of randomization and blinding raises the possibility of expectation-related influences on reported outcomes. Additionally, while ozone dosing and corticosteroid preparation were standardized within our institution, these protocols may vary across centers, which could affect reproducibility. An important consideration is the difference in treatment exposure between groups. Patients in the ozone group received three injections over three weeks, whereas those in the corticosteroid group received a single injection. This difference in cumulative procedural exposure and provider–patient interaction may have contributed to the observed outcomes. Therefore, it remains unclear whether the greater and more durable improvements observed with ozone reflect its specific biological effects or the cumulative impact of repeated interventions. Moreover, the follow-up period was restricted to 6 months. Longer-term evaluations are needed to determine the durability of treatment effects. Furthermore, treatment allocation was influenced by routine clinical practice, patient preference, and insurance coverage, as ozone therapy required out-of-pocket payment. Patients opting for ozone may have had higher expectations or motivations, potentially affecting subjective outcomes such as pain and quality of life. Although baseline characteristics were comparable, residual confounding and the influence of financial accessibility cannot be excluded. Additionally, the requirement of at least 75% pain reduction following diagnostic SIJ injection likely selected a subgroup with highly responsive and clearly defined SIJ-related pain. Although this strengthens internal validity and diagnostic accuracy, it may limit generalizability to patients with less clearly defined or multifactorial low back pain commonly seen in routine clinical practice. Therefore, the findings of this study may be most applicable to patients with strongly confirmed SIJ-mediated pain, and caution is warranted when extrapolating results to broader chronic low back pain populations. Lastly, due to the multicenter and retrospective nature of the study, strict random sampling could not be fully ensured, which should be considered when interpreting the findings. Also, potential sex-related differences could not be evaluated due to the limited sample size, and future studies with larger cohorts are warranted to address this issue.

The findings of this study suggest that ozone therapy may represent a clinically meaningful alternative for patients with sacroiliac joint dysfunction, particularly in cases where long-term symptom control and reduced corticosteroid exposure are desired. From a clinical perspective, incorporating ozone injection into interventional pain management strategies may help reduce treatment burden and improve patient-reported outcomes. Future investigations should focus on long-term outcomes, optimal dosing strategies, cost-effectiveness analyses, and the identification of patient subgroups most likely to benefit from ozone therapy. Additionally, mechanistic studies exploring the biological pathways underlying ozone’s sustained effects would further strengthen the evidence base for its clinical application.

## 5. Conclusions

This multicenter comparative study demonstrates that ultrasound-guided ozone (O_2_–O_3_) injection provides more effective and longer-lasting clinical benefits than corticosteroid injection in patients with SIJ dysfunction. While both interventions resulted in significant short-term improvements in pain, disability, and quality of life, the therapeutic effects of corticosteroids diminished by 6 months, whereas ozone therapy maintained meaningful benefits across all outcome measures. These findings suggest that ozone therapy represents a promising minimally invasive alternative to corticosteroid injections for the management of SIJ-related chronic low back pain, particularly for patients requiring longer-lasting symptom control or for whom repeated steroid exposure is undesirable. Future prospective randomized controlled trials with longer follow-up periods are warranted to further validate these findings and optimize treatment protocols.

## Figures and Tables

**Table 1:** Demographic characteristics of the study groups.

Characteristics	Corticosteroid Group (n = 31)	Ozone Group (n = 33)	*p* Value
Age (years)	44.84 ± 6.64	45.15 ± 8.60	0.872
Gender (female/male), n (%)	21 (67.7)/10 (32.3)	21 (63.6)/12 (36.4)	0.730
BMI (kg/m^2^)	28.55 ± 2.41	28.12 ± 2.16	0.460
Duration of symptoms (months)	12.0 (IQR 8)	10.0 (IQR 7)	0.471
Marital status, n (%)	Married 28 (90.3)/Single 2 (6.5)/Widowed 1 (3.2)	Married 27 (81.8)/Single 3 (9.1)/Widowed 3 (9.1)	0.750 *

Data are presented as mean ± SD or median (IQR) for continuous variables, and number (%) for categorical variables. Independent samples *t*-test or Mann–Whitney U test was used for continuous variables, as appropriate. Chi-square test or * Fisher’s exact test was used for categorical variables, as appropriate.

**Table 2:** Clinical outcomes over time in the corticosteroid and ozone groups (mean ± SD).

Outcome	Time Point	Corticosteroid Group (n = 31) Mean ± SD	Ozone Group (n = 33) Mean ± SD	*p* Value (Between Groups) *
NRS	Baseline (0 month)	7.61 ± 1.15	7.48 ± 1.42	0.693
	3rd month	4.23 ± 0.81	2.64 ± 1.22	<0.001
	6th month	7.45 ± 1.03	4.64 ± 1.32	<0.001
ODI	Baseline (0 month)	24.87 ± 4.51	27.61 ± 6.41	0.054
	3rd month	14.65 ± 3.29	11.48 ± 3.76	<0.001
	6th month	24.81 ± 4.09	18.76 ± 4.17	<0.001
SF-12 PCS	Baseline (0 month)	28.88 ± 3.27	29.10 ± 3.47	0.803
	3rd month	35.11 ± 3.64	39.75 ± 3.52	<0.001
	6th month	28.91 ± 3.54	33.15 ± 3.29	<0.001
SF-12 MCS	Baseline (0 month)	28.83 ± 5.45	29.70 ± 5.14	0.515
	3rd month	35.04 ± 5.36	38.13 ± 4.60	0.016
	6th month	28.70 ± 5.42	32.92 ± 4.79	0.002

* *p* values for between-group differences were derived from two-way repeated-measures ANOVA (group × time), following assessment of normality (Shapiro–Wilk test) and homogeneity of variances (Levene’s test). Bonferroni-adjusted post hoc (simple effects) comparisons were applied when appropriate.

**Table 3:** Within-group changes in clinical outcomes from baseline.

Outcome	Group	Change 0–3 Months, Mean Difference (95% CI)	*p* Value (Within Group) *	Change 0–6 Months, Mean Difference (95% CI)	*p* Value (Within Group) *
NRS	Corticosteroid	−3.39 (−3.87 to −2.91)	<0.001	−0.16 (−0.48 to 0.15)	0.605
	Ozone	–4.85 (–5.41 to –4.29)	<0.001	–2.85 (–3.40 to –2.30)	<0.001
ODI	Corticosteroid	–10.2 (–11.7 to –8.8)	<0.001	–0.1 (–0.7 to 0.5)	1.000
	Ozone	−16.12 (−18.95 to −13.29)	<0.001	−8.85 (−10.80 to −6.90)	<0.001
SF-12 PCS	Corticosteroid	6.22 (5.01 to 7.44)	<0.001	0.02 (−0.62 to 0.67)	1.000
	Ozone	10.66 (9.04 to 12.27)	<0.001	4.05 (2.99 to 5.12)	<0.001
SF-12 MCS	Corticosteroid	6.20 (5.50 to 6.90)	<0.001	−0.13 (−0.46 to 0.73)	1.000
	Ozone	8.43 (6.94 to 9.92)	<0.001	3.22 (2.55 to 3.89)	<0.001

* Within-group changes were analyzed using repeated-measures ANOVA, following assessment of normality (Shapiro–Wilk) and sphericity (Mauchly’s test). When sphericity was violated, Greenhouse–Geisser correction was applied.

## Data Availability

The data that support the findings of this study are available from the corresponding authors upon reasonable request.

## Abbreviations

The following abbreviations are used in this manuscript:

SIJ	Sacroiliac joint
ODI	Oswestry Disability Index
LBP	Low back pain
O_2_–O_3_	Oxygen ozone
VAS	Visual analogue score
SF-12	Short Form-12 Health Survey
FABER	Flexion, Abduction and External Rotation
CS	Corticosteroid
O	Ozone
US	Ultrasound
PRF	Platelet-rich fibrin
COX-2	Cyclooxygenase-2
WOMAC	Western Ontario and McMaster Universities Arthritis Index
NRS	Numeric rating scale
